# Posterior pericardiotomy and the prevention of post-operative atrial fibrillation and cardiac tamponade in isolated coronary artery bypass grafting – A retrospective analysis

**DOI:** 10.1186/s13019-024-02569-2

**Published:** 2024-04-24

**Authors:** Ayeshmanthe Rathnayake, Siew SC Goh, Carmel Fenton, Ashutosh Hardikar

**Affiliations:** Department of Cardiothoracic Surgery, Royal Hobart Hospital, 38 Liverpool Street, Hobart, TAS 7000 Australia

**Keywords:** Coronary artery bypass grafting, Posterior pericardiotomy, Post-operative Atrial Fibrillation, Cardiac Tamponade

## Abstract

**Background:**

Post-Operative Atrial Fibrillation (POAF) is the most frequent complication of cardiac surgery and is associated with reduced survival, increased rates of cognitive changes and cerebrovascular accidents, heart failure, renal dysfunction, infection, length of stay and hospital costs. Cardiac tamponade although less common, carries high morbidity and mortality. Shed mediastinal blood in the pericardial space is a major source of intrapericardial oxidative stress and inflammation that triggers POAF. The utilisation of a posterior pericardiotomy (PP) aims to shunt blood from pericardium into the pleural space and have a role in the prevention of POAF as well as cardiac tamponade.

**Methods:**

2168 patients had undergone isolated Coronary Artery Bypass Grafting at Royal Hobart Hospital from 2008 to 2022. They were divided into PP group vs. control group. Patient baseline demographics, intraoperative data and post-operative outcomes were reviewed retrospectively.

**Results:**

Total incidence of new POAF and cardiac tamponade was 24% and 0.74% respectively. Primary outcome of both the incidence of POAF (20.2% vs. 26.3%, *p* < 0.05) and Cardiac Tamponade (0% vs. 1.1%, *p* < 0.05) were less in the pericardiotomy group. A subgroup analysis of patients with recent myocardial infarction showed reduced incidence of POAF in the PP group (*p* < 0.05). Increasing age, Body Mass Index, poor left ventricular ejection fraction (EF < 30%) and return to theatre were independent predictors of developing POAF. There were similar rates of return to theatre for bleeding however, no cases of tamponade in the pericardiotomy group. There were no complications attributable to left posterior pericardiotomy and the time added to the duration of surgery was minimal.

**Conclusion:**

Posterior pericardiotomy is associated with a significant reduction in the incidence of POAF and cardiac tamponade which is safe and efficient.

## Background

Post-Operative Atrial Fibrillation (POAF) is the most frequent type of secondary atrial fibrillation and the most frequent complication of cardiac surgery (19–30%) and occurring more commonly after valve or concomitant surgery especially within the first 48 hours [1]. It is associated with reduced survival, increased rates of cognitive changes and Cerebrovascular Accident (CVA), heart failure, renal dysfunction, infection, and length of stay (LOS) and hospital costs [2].

Pathogenesis of atrial fibrillation after cardiac surgery is multi-factorial with identified risk factors of advanced age, hypertension, obesity, acute myocardial infarction (MI) and valvular heart disease, enlargement of the left atrium, left ventricular dysfunction, peri-atrial fat volume and electrolyte imbalance [1–3]. Potential triggers include ischemia reperfusion injury, activation of the sympathetic nervous system, systemic inflammatory response to cardiopulmonary bypass and direct trauma to right atrium during cannulation [3]. Shed mediastinal blood in the pericardial space is a major source of intrapericardial oxidative stress and inflammation that triggers POAF [4]. First introduced in 1995, Posterior Pericardiotomy (PP) has been hypothesized to reduce the incidence of POAF and cardiac tamponade by shunting blood from the pericardial space into the left pleural space [5, 6].

There have been multiple randomised controlled trials (RCTs) and recent meta-analysis reporting significantly reduction in the incidence of POAF and pericardial effusion [1, 7–10]. However, PP remains underused despite its benefits and safety profile. This observational study aims to provide further supporting data for the use of PP in isolated CABG surgery and more specifically in patients with recent MI.

## Methods

The aim of this retrospective cohort analysis was to compare the use of PP against no PP with rates of POAF and cardiac tamponade in patients undergoing isolated coronary artery bypass grafting. The Society of Thoracic Surgeons (STS) describes POAF as atrial fibrillation post-surgery requiring treatment while Heart Rhythm Society for clinical diagnosis specifies it as POAF treatment with rate or rhythm control agents, anticoagulation, and or extending hospital stay [7]. Cardiac Tamponade was diagnosed on Transthoracic Echocardiogram/ Transoesophageal Echocardiogram (TTE/ TOE) or according to operative notes during an emergency return to theatre (RTT). 2168 patients had undergone isolated CABG at Royal Hobart Hospital (RHH) from 2008 to 2022. They were divided into PP group with a single surgeon and control group with three surgeons. Patient baseline demographics, intraoperative data and post-operative outcomes were reviewed retrospectively from prospectively collected data from the Perfusion Down Under (PDU) database. The statistical analysis was performed using SPSS statistical software. All data are expressed as mean with standard deviation (SD) or number (n) and percentage (%). Categorical data are compared with Fisher’s exact test. Continuous variables between control and pericardiotomy groups were compared using Student t test. Univariate and Multivariate logistic regression model was used to predict the risk factors for developing post-operative atrial fibrillation. Odds ratio and 95% confidence interval (CI) were reported, and statistical significance was assumed at *P* < 0.05.

### Technique

A 4 cm longitudinal incision was made using cautery parallel and posterior to the left phrenic nerve, extending from the left inferior pulmonary vein to the diaphragm. Chest drains were positioned in the left hemi-thorax via anterior subxiphoid tunnelled through a separate window in the left pleura directed towards the costo-phrenic angle and in the anterior mediastinum with two additional holes made. Patients in the control arm had a total of three chest drains including the left pleura, pericardial and retrosternal with no additional holes. PP was not performed in patients with left pleural adhesions but was performed in cases of re-do surgery with cardiac adhesions only.

### Post-operative management

Criteria for chest drain removal was standard across both groups of less than 200mls in twenty-four hours. Beta-blockers (metoprolol if left ventricular ejection fraction > 50%, bisoprolol or carvedilol if left ventricular ejection fraction < 50%) were administered to all patients depending on their haemodynamic parameters and the dosage was adjusted accordingly. Potassium level was maintained > 4.5 mmol/l and magnesium maintained > 1.0mmol/l for all post-operative patients. The antithrombotic/ anticoagulation protocol was similar in both groups with aspirin and subcutaneous heparin surgical prophylaxis and commencing a second antiplatelet post-operative day three after removal of pacing wires in patients with non-ST-elevation myocardial infarction (NSTEMI), diffuse disease or endarterectomy.

## Results

**Fig. 1:**
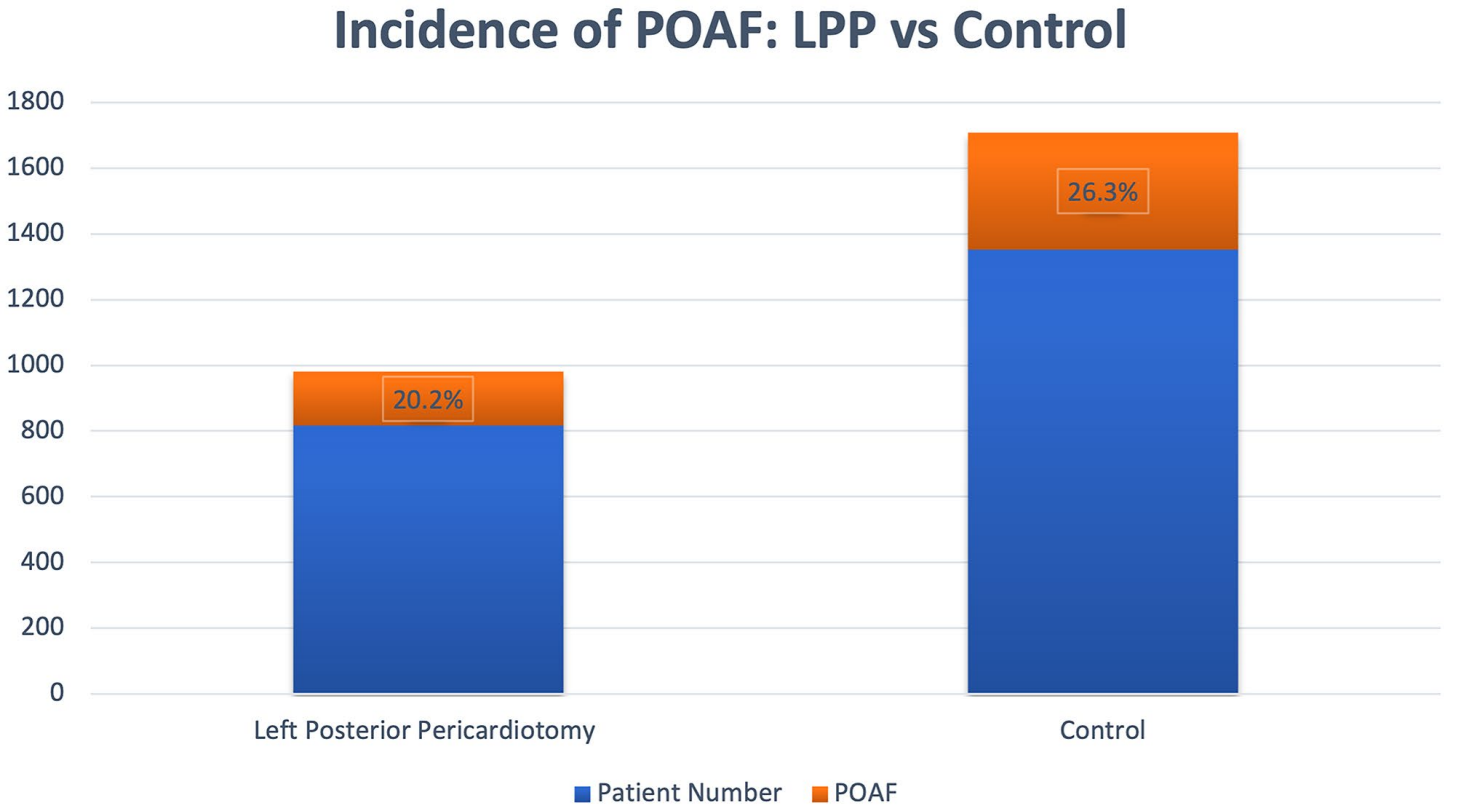
Incidence of POAF – LPP vs. Control

A total of 2168 patients underwent isolated CABG at the RHH from 2008 to 2021. 816 patients were in the PP group and 1352 patients in the control group. Baseline demographics were similar in both arms *(*Table 1*).* There was no difference in 30-day mortality, new stroke, chest drain output within the first 4 h or return to theatre for bleeding. The incidence of POAF in all patients who underwent isolated CABG was 24.03% *(*Table 2*).* The incidence POAF when comparing PP group vs. control group was 20.20% vs. 26.30% respectively in favour of the treatment group (*P* Value < 0.05 OR 1.3 95% CI 1.06 to 1.60) *(*Fig. 1*).* A subgroup analysis demonstrated a reduction in the incidence of POAF in patients in the PP arm who had a recent myocardial infarction (*P* Value < 0.05, *see* Table 3). In our cohort, increasing age, recent myocardial infarction, a higher Euroscore II, low ejection fraction and RTT for bleeding increased the risk of post-operative atrial fibrillation in univariate analysis *(see* Table 4*).* In multivariate analysis, increasing age, BMI, poor left ventricular function (EF < 30%) and return to theatre for bleeding were predictors of developing post-operative atrial fibrillation. PP had a protective effect reaching statistical significance (*P* Value < 0.05).

**Table 1:** Baseline demographics

	LPP	Control	*P* Value
Patient Number (n)	816	1352	-
Age - Years (mean ± sd)	65.93 ± 10	66.16 ± 10	0.38
BMI - kg/m2 (mean ± sd)	29.27 ± 5	29.44 ± 5	0.21
Male No. (%)	664 (81.4)	1116 (82.5)	0.83
MI - No. (%)	433 (53)	667 (49.33)	0.34
Recent MI - No. (%)	343 (42)	546 (40.38)	0.63
Hypertension - No. (%)	634 (77.7)	1074 (79.44)	0.74
Diabetes - No. (%)	238 (29.2)	417 (30.84)	0.55
Renal Impairment - No. (%)	283 (34.7)	541 (40)	0.095
Ex-smoker – No. (%)	522 (64)	838 (62)	0.65
Euroscore II			
0–4 (Low risk) – No. (%)	481 (58.94)	824 (60.95)	0.29
4–8 (Medium Risk) – No. (%)	213 (26.10)	312 (23.07)	0.22
≥ 8 (High risk) – No. (%)	122 (14.95)	216 (15.98)	0.59
LVEF- No. (%)			
> 30%	782	1313	0.83
< 30%	34	39	0.12

LPP: Left Posterior Pericardiotomy BMI: Body Mass Index. MI: Myocardial Infarction. LVEF: Left Ventriclar Ejection Fraction

**Table 2:** Operative data

	LPP	Control	*P* Value
On-PUMP – No. (%)	807 (98.9)	1333 (98.6)	0.96
Off-PUMP – No. (%)	9 (1.1)	19 (1.4)	0.55
Urgency of Surgery			
Elective – No. (%)	514 (63)	855 (63)	0.96
Urgent – No. (%)	286 (35)	470 (35)	0.93
Emergent/ Salvage – No. (%)	16 (2)	27 (2)	0.95
Number of Grafts – No. (%)			
< 3	106 (13)	135 (10)	0.06
≥3	710 (87)	1217 (90)	0.60
Chest Drain Output Initial 4 h – mls (mean ± STD)	285 ± 165	316 ± 192	0.15
30d Mortality - No. (%)	7(0.86)	10(0.74)	0.77
New Stroke - No. (%)	7 (0.86)	19 (1.4)	0.26
New Arrhythmia - No. (%)	196 (24.0)	396 (29.3)	< 0.05
New Atrial Fibrillation - No. (%)	165 (20.2)	356 (26.3)	< 0.05
Cardiac Tamponade	0	16	< 0.05
Return to Theatre for Bleeding	15 (1.84)	34 (2.51)	0.52
Mean LOS - Days	7.14	7.56	0.22

LOS: Length of Stay

**Table 3:** Subgroup analysis – PP and POAF in patients with recent MI

	PP	Control	*P* Value
Number – n (%)	343 (42.03)	546 (40.38)	0.63
POAF – n (%)	66 (19.24)	147 (26.92)	< 0.05

**Table 4:** Univariate analysis of risk factors predisposing to POAF

	POAF (*n* = 521)	Non-POAF (*n* = 1647)	*P* Value
PP	165	651	< 0.05
Age	69 ± 9	64 ± 10	< 0.05
Recent MI	311 (60)	578 (35)	< 0.05
Euroscore II – mean ± sd	4.3 ± 5	3.4 ± 4	< 0.05
LVEF < 30% – no. (%)	28 (5.4)	45 (2.7)	< 0.05
RTT for Bleeding – No. (%)	18 (3.5)	31 (1.9)	< 0.05
BMI - kg/m2 (mean ± sd)	29 ± 5	29 ± 5	0.2
Hypertension	453 (87)	1255 (76)	0.08
COPD	41(8)	132 (8)	0.92
Diabetes	157 (30)	498 (30)	0.97

COPD: Chronic Obstructive Pulmonary Disease

Return to Theatre (RTT) for bleeding occurred predominantly within twenty-four hours and less than 5% of cases occurring later. Although the incidence of RTT for bleeding in both groups were similar there were no cases of cardiac tamponade in the PP group (*P* Value < 0.05). Furthermore, there were no cases of cardiac or graft herniation, thermal injury to the phrenic nerve as well as no significant difference in left-sided pleural effusions requiring inter-costal catheter insertion or subsequent pulmonary complications.

## Discussion

This study retrospective cohort analysis found that patients with PP undergoing isolated CABG surgery had a significantly lower incidence of POAF and cardiac tamponade. This in keeping with previous meta-analyses, RCTs and majority of retrospective cohort studies [1, 6, 7].

The difference in POAF incidence between the two groups was 6.1% and represents one of the largest cohort studies specifically comparing PP in patients undergoing isolated CABG. It is important to consider although majority of baseline demographics were similar, there was a slightly higher proportion of recent myocardial infarction and lower ejection fraction in the PP group thus a potential confounding factor. This may translate to a higher risk population in addition to recent antiplatelet therapy thus a higher propensity for post-operative bleeding events. 30-day mortality was slightly more in the treatment group (0.86% vs. 0.74% *P* value 0.77) which can be explained by a higher risk group however, a subgroup analysis demonstrates benefit of PP within patients with recent myocardial infarction and POAF. This could be explained by the relation between the use of pre-operative beta-blockers and higher rates of new-onset atrial fibrillation given majority of these patients would be commenced on beta-blockers as an inpatient. Additionally, many of these patients were commenced on dual antiplatelet therapy thus may contribute to the burden of shed mediastinal blood if not diverted away from the pericardial space via use of PP.

It can be derived from the similar rates of return to theatre for bleeding between the two groups that there is a protective factor for cardiac tamponade with no cases reported in the PP group. This can be beneficial for both patient and surgeon in the context of not requiring emergent re-opening within the intensive care unit and allow more time for a controlled re-opening and exploration in the operating theatre providing an optimal technical and sterile setting. The sequelae of re-opening in an emergency setting can be avoided thus reduce the risk of sternal wound complications and the associated morbidity and mortality. There was not a statistically significant result with regards to CT within the subgroup analysis of patients with recent myocardial infarction. This can be explained by inadequate numbers of affected patients given there was an overall benefit when comparing the entire cohort.

It is important to consider opposing literature where several individual articles have shown no significant differences in the incidence of POAF and PP [1, 11–14]. However, further analysis of most of these articles demonstrate a significant reduction of pericardial effusions and a correlation with a lower incidence of POAF in patients with mild-moderate vs. large pericardial effusions [11–13]. Left pleural effusions requiring post-operative drainage and pulmonary complications with PP is an important consideration and the variability in literature is reflective of potential confounding factors. The most recent meta-analysis did demonstrate a higher incidence of left pleural effusions in the PP group but there was no increased risk of pulmonary complications [1]. Our data is in keeping with the PALACS Trial with no significant difference in left pleural effusions or other known complications including but not limited to thermal phrenic nerve injury or cardiac herniation [1].

This is a retrospective cohort study and is open to selection bias, missing records and/or misclassification of data. The PDU Databases are standardized and accurate data collection tools thus mitigating potential errors and missing data. This was a single-centre study and only a single surgeon performs this technique with the supervised fellows and trainees only encompassing a small percentage of the total cohort. Ideally a multicentred study with randomization would allow for more robust results however, our results are in keeping with previous RCTs and metanalyses. Therefore, this study provides additional support in the use of PP in isolated CABG and especially in patients with recent MI as a starting point.

## Conclusions

PP is a safe and useful technique in the prevention of POAF and CT in isolated CABG. Utilising PP in patients with recent myocardial infarction is beneficial. Although it does not contribute to randomized evidence, there is a large body of current literature supporting the underused technique of PP and the findings of this observational study is in support of its routine use in isolated CABG.

## Data Availability

The datasets used and/or analysed during the current study are available from the corresponding author on reasonable request.

## Abbreviations

POAF: Post–Operative Atrial Fibrillation
LOS: Length of Stay
RHH: Royal Hobart Hospital
CABG: Coronary Artery Bypass Grafting
BMI: Body Mass Index
EF: Ejection Fraction
CVA: Cerebrovascular Accident
PP: Posterior Pericardiotomy
RCT: Randomised Controlled Trial
ECG: Electrocardiography
TTE: Transthoracic Echocardiogram
TOE: Transoesophageal Echocardiogram
PDU: Perfusion Down Under
RTT: Return to Theatre
